# Radiological Manifestations of Lymphangioleiomyomatosis: A Local Patient Cohort Analysis

**DOI:** 10.7759/cureus.87292

**Published:** 2025-07-04

**Authors:** Luca Conti, Darlene Mercieca, Gianluca Gatt, Luca Sant Fournier, Peter Fsadni

**Affiliations:** 1 Respiratory Medicine, Mater Dei Hospital, Birkirkara, MLT

**Keywords:** angiomyolipoma, cystic lung disease, lymphangioleiomyomatosis, sporadic, tuberous sclerosis complex

## Abstract

Background: Lymphangioleiomyomatosis (LAM) is an ultra-rare neoplastic cystic disease primarily affecting females of reproductive age, characterized by the infiltration of smooth muscle cells into the lungs and the formation of cystic lesions. This study aims to compare the radiological findings in patients with sporadic LAM (s-LAM) and those with LAM associated with tuberous sclerosis complex (TSC-LAM) in the Maltese population.

Methods: A retrospective observational study was conducted at Mater Dei Hospital, Malta, involving eight patients diagnosed with LAM between 2014 and 2024. Thoracic and abdominal CT scans were reviewed, and findings were categorized based on the presence of s-LAM or TSC-LAM.

Results: The cohort included five patients with s-LAM and three with TSC-LAM, alongside 15 patients with tuberous sclerosis complex (TSC) but no LAM. All LAM patients exhibited diffuse thin-walled cysts, with sizes ranging from 7 to 72 mm. Notably, chylous pleural effusions were observed only in the s-LAM group. The TSC-LAM group demonstrated significant extra-pulmonary manifestations, including renal angiomyolipomas, with some requiring intervention.

Conclusion: This study highlights the distinct radiological features and complications associated with s-LAM and TSC-LAM. The findings underscore the necessity for vigilant screening and monitoring in TSC patients to manage potential LAM-related complications effectively. A multidisciplinary approach is essential for optimizing patient outcomes and addressing the broader implications of TSC and LAM on health.

## Introduction

Lymphangioleiomyomatosis (LAM) is a rare neoplastic cystic disease that falls under the category of PEComas, which are mesenchymal tumors made up of unique perivascular epithelioid cells (PECs) that can be identified through histological and immunohistochemical methods. The disease is marked by the infiltration of smooth muscle cells of unknown origin into the lungs, leading to the formation of cystic lesions or tumors known as angiomyolipomas (AMLs), typically located in the kidneys or involving the lymphatic vessels, resulting in lymphangioleiomyomas [1]. LAM predominantly affects females of reproductive age and is exceptionally rare in males and children. The initial estimated prevalence of LAM was three to seven cases per million women, though recent studies show this is grossly underestimated. The European prevalence is 23.5 cases per million adult females and 19 cases per million when considering the total living female population of all ages [2]. It often occurs sporadically, affecting approximately one in every 400,00 adult females, or can be associated with tuberous sclerosis complex (TSC), an autosomal dominant condition characterized by the development of hamartomatous lesions in various organs. A total of 30-40% of TSC patients may develop LAM [3]. The purpose of this study is to detail and compare the array of radiological findings in our cohort of sporadic LAM (s-LAM) and LAM associated with tuberous sclerosis complex (TSC-LAM) patients for the first time in the Maltese population and review the current literature.

## Materials and methods

This is a single-center, retrospective, observational study conducted on patients who obtained a final diagnosis of LAM between 2014 and 2024 at Mater Dei Hospital, Malta. We performed a retrospective review of radiological imaging of 23 patients identified. LAM was diagnosed according to the European Respiratory Society [3] and the American Thoracic Society [4] guidelines: five patients had s-LAM, of which four had definite s-LAM and one had probable s-LAM, and three had TSC-LAM. The remaining 15 patients had evidence of TSC but no LAM. Pulmonary and extra-organ involvement findings were recorded in all patients, and a comparison was made between the s-LAM and TSC groups.

Detailed documentation of the pulmonary and extra-pulmonary manifestations is valuable in determining prognosis, monitoring treatment effects, and managing complications. Through this retrospective observational study, TSC patients who are not followed up for these potential complications will be identified, thus aiming to reduce morbidity and mortality.

Statistical analysis

In this study, various statistical analyses were employed. Continuous variables were evaluated for normality and reported as mean ± standard deviation when normally distributed. Categorical variables were summarized as frequencies and percentages. To compare categorical outcomes, appropriate statistical tests were applied, including the ANOVA test, t-test, chi-squared test, and Fisher’s exact test; the latter was used when sample sizes were small. A p-value of less than 0.05 was considered statistically significant.

## Results

The TSC-LAM and s-LAM groups consisted entirely of female patients with a mean age of 47.25 ± 11.2 years (range = 33-71) (Table 1). A further 15 patients, including 10 males and five pre-menopausal females, were identified as suffering from tuberous sclerosis with no evidence of LAM. Two patients from the TSC-LAM group were not referred to respiratory physicians and did not have a dedicated high-resolution computed tomography (HRCT) scan. Diagnosis was reached in these patients based on an incidental finding on abdominal imaging; in one patient dating back to 2011. Four (26.7%) females suffering from TSC without LAM did not have any chest imaging performed within the last 10 years.

**Table 1:** Baseline demographic and clinical characteristics of the patients. * Plus-minus values are means ± SD. ^#^ Race was self-reported. § P-value was calculated with the use of the one-way ANOVA test. The ANOVA F-value is 0.066. ​​​​​​​^**^ P-value was calculated with the use of the chi-squared test. The chi-squared statistic is 3.764. ​​​​​​​^‡^ P-value was calculated with the use of Fisher's exact test. TSC: tuberous sclerosis complex; LAM: lymphangioleiomyomatosis; s-LAM: sporadic lymphangioleiomyomatosis; TSC-LAM: lymphangioleiomyomatosis associated with tuberous sclerosis complex.

Characteristic	s-LAM (N = 5)	TSC-LAM (N = 3)	TSC no LAM (N = 15)	p-value
Sex				
Female	5 (100)	3 (100)	5 (33.3)	
Age, years	44.3 ± 4.8^*^	49.0 ± 13.5^*^	42.5 ± 13.7^*^	0.94^§^
Race, No. (%)^#^				
White Caucasian	4 (80)	3 (100)	15 (100)	0.152^**^
Asian	1 (20)	0	0	
Clinical features, No. (%)				
Pulmonary nodules	0 (0)	1 (33.3)	3 (20)	0.375^‡^
Chylous pleural effusions	1 (20)	0 (0)	0 (0)	0.25^‡^
Pneumothorax	0 (0)	0 (0)	0 (0)	1^‡^
Renal angiomyolipomas	1 (20)	3 (100)	8 (53.3)	0.143^‡^
Liver angiomyolipoma	0 (0)	1 (33.3)	4 (26.7)	0.375^‡^
Lymphangioleiomyomas	2 (40)	0 (0)	0 (0)	0.053^‡^
Chylous ascites	0 (0)	0 (0)	0 (0)	1^‡^
Sclerotic bone lesions	0 (0)	0 (0)	4 (26.7)	0.53^‡^
Cardiac rhabdomyomas	0 (0)	0 (0)	2 (13.3)	1^‡^

**Figure 1:**
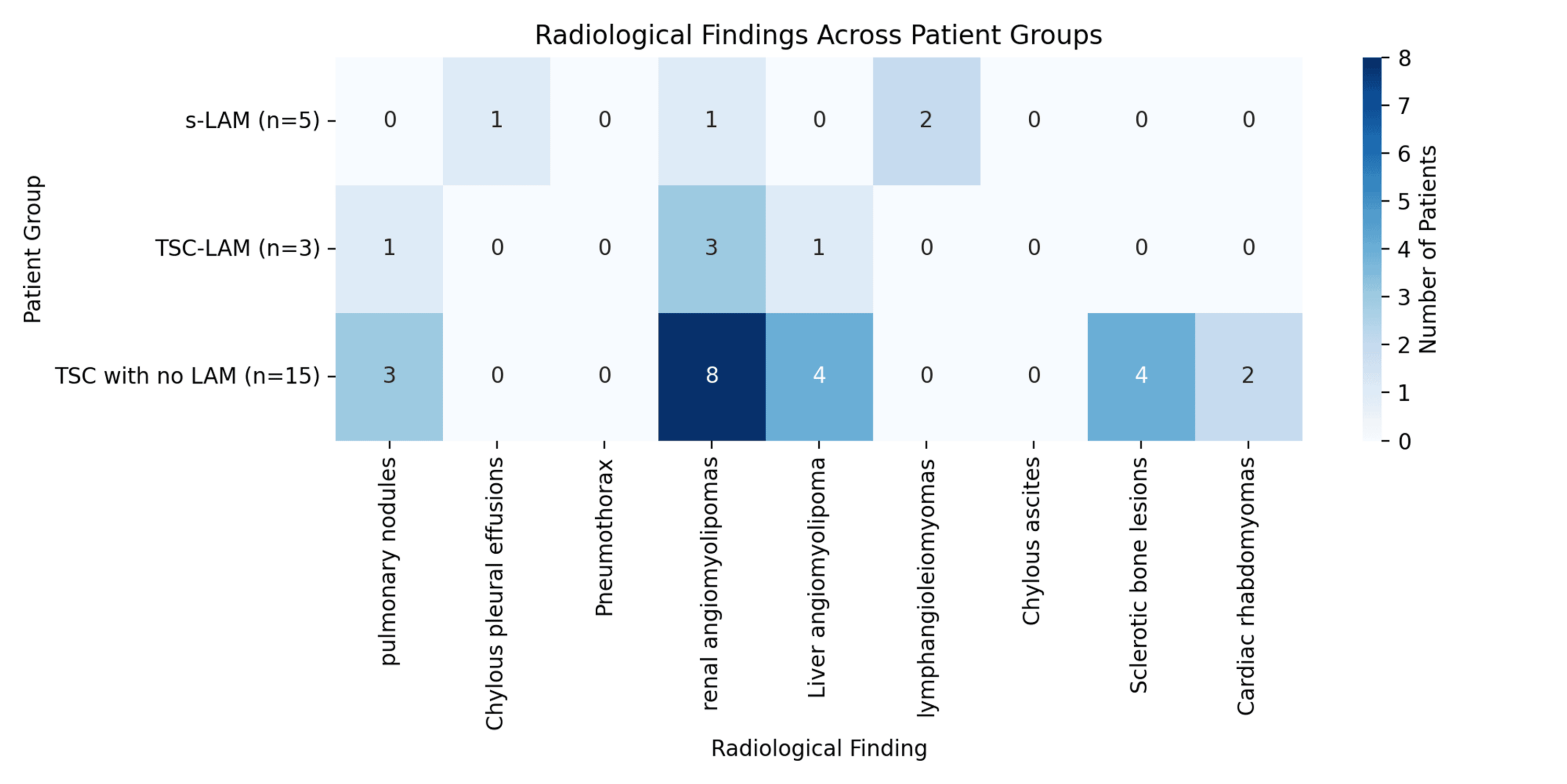
A heatmap showing the distribution of radiological findings across the three patient groups. Darker shades indicate a higher number of patients with that finding in the respective group. TSC: tuberous sclerosis complex; LAM: lymphangioleiomyomatosis; s-LAM: sporadic lymphangioleiomyomatosis; TSC-LAM: lymphangioleiomyomatosis associated with tuberous sclerosis complex.

The numerous clinical findings are represented in Figure 1 and Table 1. None of the findings reached statistical significance, though the presence of lymphangioleiomyomas approached significance (p-value = 0.053), yet still not statistically significant. All other findings had higher p-values, indicating no statistically significant differences between the groups for those features in the dataset.

**Figure 2:**
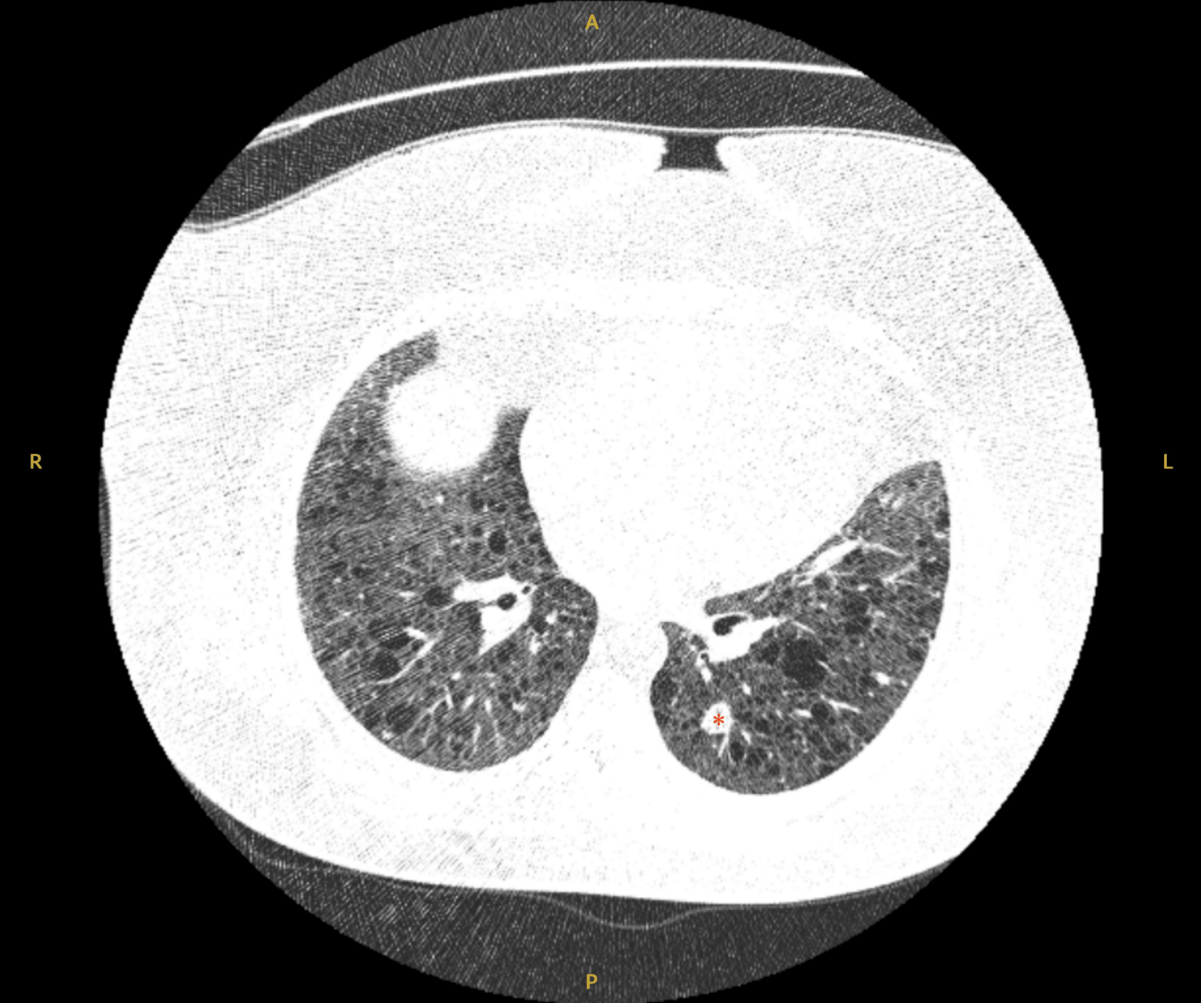
Cross-sectional axial high-resolution computed tomography image showing evidence of diffuse thin-wall cysts as well as a 22-mm multifocal micronodular pneumocyte hyperplastic lesion in the left lower lobe (*). A: anterior; P: posterior; R: right; L: left.

Diffuse thin-walled cysts were present in all LAM patients, and maximum cyst sizes ranged from 7 to 72 mm. Distribution was uniform throughout the lungs without zonal predominance. Multiple pulmonary nodules, likely representing multifocal micronodular pneumocyte hyperplasia (MMPH), are usually seen in association with TSC but may occur with or without concurrent LAM. In our cohort, this was identified in one (33.3%) TSC-LAM patient, with the largest measuring 22 mm (Figure 2), and in three (20%) TSC with no LAM patients. Chylous pleural effusions were only identified in one (20%) s-LAM patient, which required recurrent pleural drainages. Pneumothoraces were not identified in either group.

**Figure 3:**
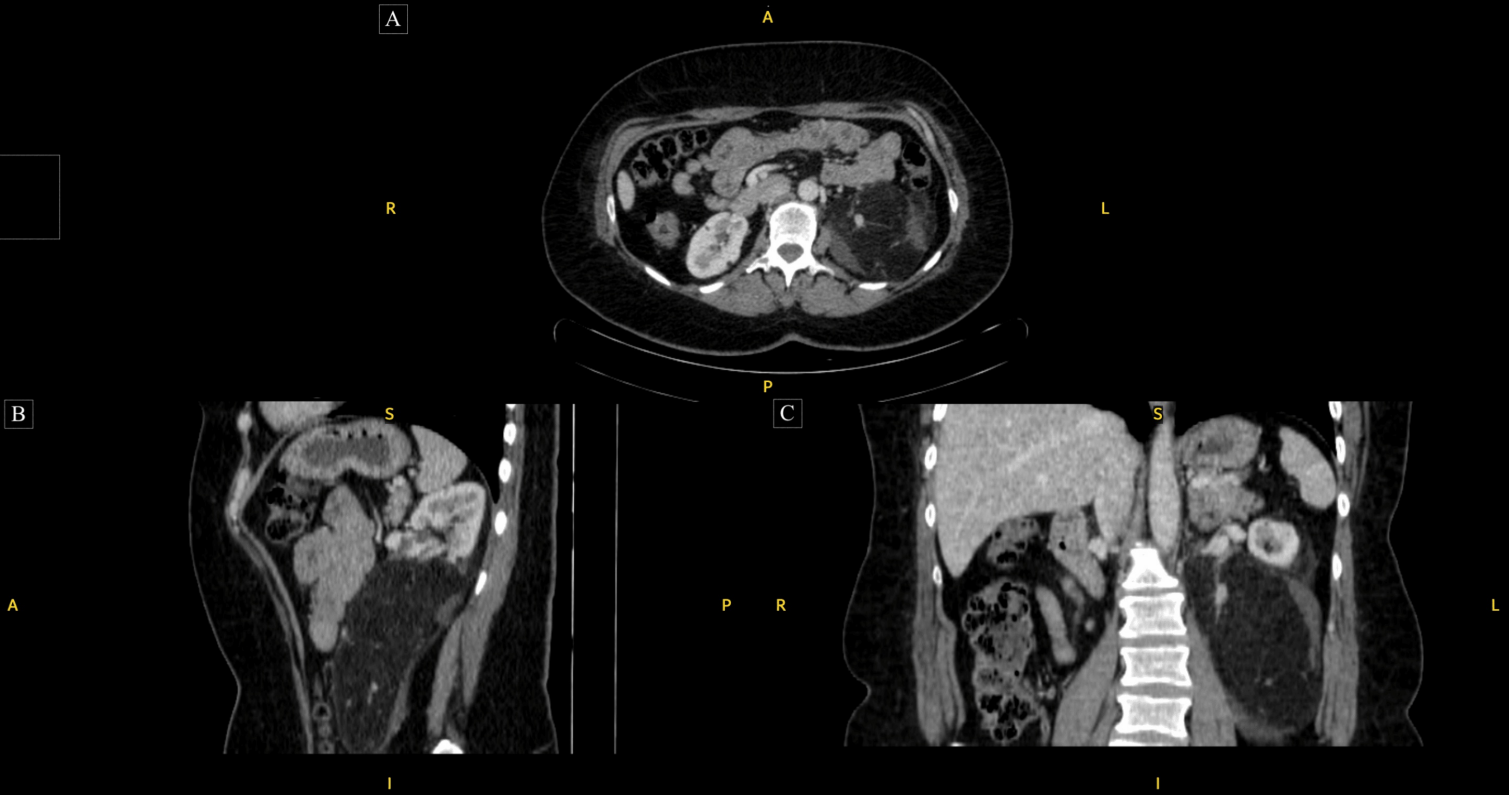
Axial (A), sagittal (B), and coronal images (C) of a large left-sided renal angiomyolipoma. A: anterior; P: posterior; S: superior; I: inferior; R: right; L: left.

Most patients with TSC also had significant extra-pulmonary manifestations, particularly renal AML, with diameters up to 160 mm (Figure 3), necessitating interventions with embolizations and partial nephrectomies. Based on the analysis of the angiomyolipoma diameter data between the three groups, angiomyolipoma diameters are significantly larger in the TSC-LAM group compared to the TSC with no LAM group (p-value = 0.006) (Table 2). As only one patient in the s-LAM had an AML, the analysis was performed between TSC-LAM (mean: 100.0 ± 54.5 mm) and TSC with no LAM (mean: 72.0 ± 22.7 mm) groups. Six patients required embolization, three (100%) patients from the TSC-LAM group and three (20%) from the TSC with no LAM group. Three patients, two (66.6%) in the TSC-LAM group and one (6.7%) in the TSC with no LAM group, required more than one session in view of hemorrhagic angiolipomas. One (20%) patient in the s-LAM group had evidence of polycystic kidney disease but no AMLs. There was no statistical relationship between the renal AML diameter and the need for intervention (p-value = 0.40), though a statistical association between initial intervention and the need for recurrent embolizations reached statistical significance (p-value = 0.015).

**Table 2:** Renal angiomyolipomas and renal interventions. * Plus-minus values are means ± SD. § P-value was calculated with the use of the one-way ANOVA test. ^** ^P-value was calculated with the use of the chi-squared test. TSC: tuberous sclerosis complex; LAM: lymphangioleiomyomatosis; s-LAM: sporadic lymphangioleiomyomatosis; TSC-LAM: lymphangioleiomyomatosis associated with tuberous sclerosis complex.

Characteristic	s-LAM (N = 5)	TSC-LAM (N = 3)	TSC no LAM (N = 15)	F value or chi-squared value	p-value
Renal AML diameter (mm)	30	100 ± 54.5^*^	72.0 ± 22.7^*^	10.27	0.006^§^
Intervention, No. (%)	0	3 (100)	3 (20)	1.83	0.4^**^
Recurrent interventions, No. (%)	0	2 (66.6)	1 (6.7)	5.86	0.015^**^

Additional organ involvement includes liver angiolipomas in about half of the TSC-LAM group, although no lymphangioleiomyomas or cardiac rhabdomyomas were noted. There is less multi-organ involvement in s-LAM, with some instances of lymphangioleiomyomas. No chylous ascites was identified in either group.

In cases of tuberous sclerosis without LAM, pulmonary abnormalities typical of LAM were absent, though only a minority of patients had an initial HRCT performed. This group also exhibited more frequent sclerotic bone lesions and echocardiogram abnormalities, likely due to the broader impact of tuberous sclerosis.

None of the spirometry parameters show statistically significant differences between the s-LAM and TSC-LAM groups (all p-values > 0.05) (Table 3). The negative t-value for the forced expiratory volume in one second (FEV1)/forced vital capacity (FVC) ratio indicates that the TSC-LAM group had a slightly higher mean ratio than the s-LAM group, though this difference is not statistically significant. Two individuals (66.6%) in the TSC-LAM group, as well as three individuals (20%) with tuberous sclerosis with no LAM, had AMLs >3 cm, suggesting the need for treatment.

**Table 3:** Pulmonary function tests. ^*^ Plus-minus values are means ± SD. ^§^ P-value was calculated with the use of the t-test. s-LAM: sporadic lymphangioleiomyomatosis; TSC-LAM: lymphangioleiomyomatosis associated with tuberous sclerosis complex; FEV1: forced expiratory volume in one second; FVC: forced vital capacity.

	s-LAM	TSC-LAM	t-value	p-value^§^
Performed, No. (%)	5 (100)	2 (66.6)	-	-
FEV1 volume, ml	2352 ± 1279^*^	1830 ± 919^*^	0.603	0.573
FEV1 % of predicted value	85.4 ± 23.7^*^	62 ± 29.0^*^	1.014	0.357
FVC volume, ml	2710 ± 1616^*^	2275 ± 1096^*^	0.957	0.383
FVC % of predicted value	80.8 ± 26.5^*^	63.5 ± 27.6^*^	1.103	0.32
Ratio of FEV1 to FVC	0.93 ± 0.09^*^	0.8 ± 0.01^*^	0.986	0.369

## Discussion

The presence of lymphangioleiomyomas approached significance (p-value = 0.053) in our study. Statistical analysis showed larger angiomyolipomas in the TSC-LAM group compared to the TSC with no LAM group (p-value = 0.006). This can be explained due to the combined effects of LAM cell involvement, enhanced mammalian target of rapamycin (mTOR) signaling, hormonal sensitivity, and possibly more aggressive tumor biology. This, therefore, reflects the more severe systemic nature of TSC-LAM compared to TSC without LAM.

There was no significant difference between the three groups and medical intervention required. A statistically significant association between initial intervention and the need for recurrent embolization was identified (p-value ≈ 0.015, p < 0.05).

In our study cohort, two individuals (66%) in the TSC-LAM group qualify for treatment but are not currently receiving it. Specifically, one with a significant AML size showing signs of interval growth and the other with a very large AML that has experienced multiple unsuccessful embolizations (Figure 3). Three individuals (20%) with tuberous sclerosis with no LAM qualify for treatment but only one patient is currently receiving treatment. There is an urgent need to address the situation of patients who meet treatment criteria but are not currently receiving treatment. This involves reviewing their cases to identify barriers to treatment and addressing these issues promptly.

The diagnosis and management of TSC and its pulmonary manifestation LAM require a comprehensive and multidisciplinary approach, given the complexity of these conditions [5]. This discussion highlights critical diagnostic inquiries, imaging protocols, and management strategies tailored to adult patients with TSC, focusing on the identification and monitoring of LAM [6].

In adult patients diagnosed with TSC, a detailed history focusing on tobacco exposure, manifestations of connective tissue diseases, and pulmonary symptoms such as dyspnea, cough, and spontaneous pneumothorax is essential [5]. These factors can significantly impact the clinical progress and management of TSC. Notably, tobacco exposure is a known risk factor for respiratory complications, and its assessment should be done routinely when assessing these patients [4].

For screening, it is recommended that baseline chest CT imaging be performed on all TSC females and symptomatic males starting at the age of 18 years. The utilization of ultra-low-dose CT protocols is advisable to minimize radiation exposure, particularly in younger patients who may require ongoing surveillance. This is crucial, as over time, cumulative radiation exposure can lead to an increased risk of secondary malignancies [5].

Patients with lung cysts consistent with LAM should undergo baseline pulmonary function tests (PFTs), including spirometry and diffusion capacity tests. These tests provide vital information regarding the functional status of the lungs and serve as baseline measurements for ongoing monitoring. The inclusion of a six-minute walk test (6MWT) and cardiopulmonary exercise testing (CPET) can further evaluate exercise capacity and symptom burden, offering a holistic view of the patient’s respiratory health [3,5].

For patients already diagnosed with LAM, tailored surveillance strategies are necessary. Asymptomatic adult females with negative screening CTs should undergo HRCT screening every five years through menopause, ensuring early detection of any emerging pulmonary complications [3]. Conversely, patients exhibiting cystic lung disease on initial screenings should have follow-up HRCTs on a case-by-case decision, with adaptations based on clinical presentation and progression [5].

Routine PFT monitoring remains a cornerstone of management for patients with diagnosed LAM, especially those showing signs of disease progression [3,5,7]. The frequency of these assessments should be intensified in patients with rapid deterioration or those undergoing therapeutic interventions. The initiation of mTOR inhibitors is warranted in cases where lung function is compromised or there is a substantial disease burden, such as large AMLs >3 cm or evidence of growing AMLs. This therapeutic approach has shown promise in stabilizing lung function and improving the quality of life for patients [7].

Regular spirometry tests are vital for monitoring lung function, particularly in patients with TSC-LAM and s-LAM, as they facilitate early detection and management of respiratory problems [1,3,5,7]. In our cohort, one patient (33.3%) in the TSC-LAM group did not undergo a spirometry test at diagnosis, suggesting that the patient was unaware of their condition and did not consult a respiratory physician. This reveals a gap in both diagnosis and management. It is crucial to implement routine spirometry tests (every six to 12 months) for all patients with TSC-LAM and s-LAM to track disease progression and adjust treatment as needed.

For patients with suspected LAM, a high-quality pulmonary HRCT scan utilizing advanced imaging techniques is essential for accurate diagnosis. Additionally, abdominal imaging is critical for identifying associated lesions such as AMLs, which are prevalent in this patient population. Routine screening for asymptomatic lymphangioleiomyomas is not recommended, emphasizing the need for a case-by-case evaluation [3].

A multidisciplinary approach encompassing genetic counseling, physical examinations, and thorough history-taking is vital for the comprehensive management of patients with LAM. This holistic strategy not only addresses the pulmonary aspects of the disease but also considers the broader implications of TSC on patients' overall health and well-being [3,6].

This local study has limitations. Apart from being retrospective and single-centered, this study is limited by its small sample size (n = 8), with uneven patient group distributions. Such small cohorts reduce statistical power, increase susceptibility to random variation and outlier effects, and limit the reliability of subgroup comparisons. The imbalance in group sizes further constrains the robustness of statistical analyses. Consequently, the findings may lack generalizability and should be interpreted with caution.

## Conclusions

In conclusion, patients with TSC and LAM require vigilant screening, regular monitoring, and personalized management strategies to optimize outcomes. This study has identified the need for increased awareness of pneumothorax risk and the importance of prompt medical attention for symptoms. Support from patient advocacy groups can alleviate feelings of isolation and provide essential resources for those navigating the complexities of these orphan diseases. As research advances, ongoing collaboration among healthcare providers will be essential to enhance care quality and improve the lives of individuals affected by TSC and LAM.
